# Chitosan-Based Films Blended with Tannic Acid and Moringa Oleifera for Application in Food Packaging: The Preservation of Strawberries (*Fragaria ananassa*)

**DOI:** 10.3390/polym16070937

**Published:** 2024-03-29

**Authors:** Raja Venkatesan, Alexandre A. Vetcher, Bandar Ali Al-Asbahi, Seong-Cheol Kim

**Affiliations:** 1 School of Chemical Engineering, Yeungnam University, 280 Daehak-Ro, Gyeongsan 38541, Republic of Korea; 2 Institute of Biochemical Technology and Nanotechnology, Peoples’ Friendship University of Russia n.a. P. Lumumba (RUDN), 6 Miklukho-Maklaya Str., 117198 Moscow, Russia; avetcher@gmail.com; 3 Department of Physics and Astronomy, College of Science, King Saud University, P.O. Box 2455, Riyadh 11451, Saudi Arabia; balasbahi@ksu.edu.sa

**Keywords:** chitosan, Moringa oleifera seed powder (MOSP), tannic acid (TA), antimicrobial and antifungal activity, shelf life food packaging

## Abstract

Biobased plastics provide a sustainable alternative to conventional food packaging materials, thereby reducing the environmental impact. The present study investigated the effectiveness of chitosan with varying levels of Moringa oleifera seed powder (MOSP) and tannic acid (TA). Chitosan (CS) biocomposite films with tannic acid acted as a cross-linker, and Moringa oleifera seed powder served as reinforcement. To enhance food packaging and film performance, Moringa oleifera seed powder was introduced at various loadings of 1.0, 3.0, 5.0, and 10.0 wt.%. Fourier-transform infrared spectroscopy, X-ray diffraction, and scanning electron microscopy analyses were performed to study the structure and morphology of the CS/TA/MOSP films. The scanning electron microscopy results confirmed that chitosan/TA with 10.0 wt.% of MOSP produced a lightly miscible droplet/matrix structure. Furthermore, mechanical properties, swelling, water solubility, optical barrier, and water contact angle properties of the film were also calculated. With increasing Moringa oleifera seed powder contents, the biocomposite films’ antimicrobial and antifungal activity increased at the 10.0 wt.% MOSP level; all of the observed bacteria [*Staphylococcus aureus* (*S. aureus*), *Escherichia coli* (*E. coli*), *Aspergillus niger* (*A. niger*), and *Candida albicans* (*C. albicans*)] had a notably increased percentage of growth. The film, with 10.0 wt.% MOSP content, effectively preserves strawberries’ freshness, making it an ideal food packaging material.

## 1. Introduction

In various fields, including aerospace and medicine, thermoplastic polymers play a crucial role [1,2]. The advantages of plastics over their metal counterparts are versatility, large-scale manufacturing, low prices, flexibility, durability, and water resistance capacity [3]. Most polymers used in food packaging are non-biodegradable, and they do not easily decompose by natural processes. This leads to plastic pollution (the accumulation of plastic waste in landfills and the environment, harming ecosystems and wildlife) and microplastics (the breakdown of plastic into tiny fragments, further increasing environmental and potential health risks) [4]. The use of biobased packaging materials as replacements for synthetic-based polymers is increasing at present [5,6].

Biobased polymers offer numerous positive aspects for environmental protection, including a reduced reliance on fossil fuels, biodegradability, a lower carbon footprint, sustainable resource utilization, and the potential to contribute to a circular economy. Biobased polymers can be divided into two main groups: synthetic and natural polymers. The most widely used types of polymers on Earth are referred to as natural polymers. Examples include cellulose [7,8], chitin/chitosan [9,10,11,12], and other macromolecules. Biobased polymers have been studied as coatings and food packaging materials because of their non-toxicity and excellent mechanical and thermal characteristics. According to the literature [13,14], natural polymers have outstanding flexibility and great potential for use in food packaging. Chitosan is an excellent choice for film production [15]. Biobased film can be functionalized with the aim of enhancing its functionality and efficiency because there are changes in chemical structure, crystallization, and barrier characteristics.

Chitosan (β-(1,4)-2-amino-2-deoxy-d-glucopyranose) is a type of biopolymer that is naturally derived from biomass. It is cationic and is considered to be biocompatible. Being a biopolymer, it is also inherently safe for use in various applications [16,17,18]. The degree of deacetylation of chitosan refers to the proportion of *N*-acetyl-D-glucosamine units that have been deacetylated to D-glucosamine. The ability of materials to adapt to multiple forms, such as powders, films, membranes, and sheets, has been carefully studied in various sectors, including water treatment, cosmetics, biomedicine, packaging, and coatings [19,20,21]. It is used in a variety of packaging materials, such as waste bags, film wraps, and meals in solid containers [22,23]. Tannic acid (TA) is a polyphenolic compound found in different plants, including fruits, seeds, and bark. TA has been investigated for its antimicrobial activity. It may disrupt bacterial cell membranes, inhibit bacterial enzymes, and interfere with bacterial adhesion [24]. TA is a cross-linking agent as it is water soluble, polyphenolic, amphiphilic, and has high cross-linking and antioxidant activities [25,26]. TA has been explored for different applications, including medicine, food packaging, and as an antimicrobial agent. However, metal and metal oxide particles are used as chemical reinforcing agents. Food packaging includes inorganic materials like SiO_2_ [27,28], TiO_2_ [29,30,31], ZnO [32,33,34,35,36], and MgO [37,38,39]. The adverse effects of the conventional method of food packaging are reduced by active packing, which blends inorganic materials with chitosan [40,41].

Moringa oleifera seed powder (MOSP), commonly known as the drumstick tree, is a plant that is native to parts of Africa and Asia. It has been traditionally used for medicinal and food packaging purposes. MOSP contains a variety of bioactive compounds, such as alkaloids, flavonoids, and phenolic acids, which have been reported to exhibit antimicrobial activity [42]. In addition to its antibacterial characterization, MOSP has anti-inflammatory and antioxidant activities. This multifaceted activity can contribute to its efficiency in bacteria. MOSP has confirmed antimicrobial activity against a wide range of bacteria, with both Gram-positive and Gram-negative strains. MOSPs have been found to have relatively low toxicity, making them potentially suitable for food packaging applications. Currently, chitosan film has been developed with starch [43,44,45], lignin [46,47,48], polylactic acid (PLA) [49,50], PBAT [51,52], and other materials [53,54,55,56,57,58]. The majority of the process of preparation for these composite materials, however, involves solution casting, which is easy to perform in the lab but difficult to scale up commercially [13,59]. The use of MOSP in food packaging is authorized by the Food and Drug Administration (FDA). A few MOSPs are utilized in the packaging used in active packaging methods as antimicrobials to extend shelf life.

Prior studies investigated the chitosan/okra powder/silicon (Lin et al., 2020), chitosan/gelatin/cinnamon essential oil/rutin [60], chitosan/gelatin/cinnamon essential oil [61], chitosan/apple peel polyphenols [62], chitosan/poly(trimethylene carbonate) [63], chitosan/Ag/ZnO [64,65,66], polyurethane/chitosan/ZnO [67], polyurethane/chitosan/ZnO [68], TiO_2_-ZnO/chitosan/graphene [69], chitosan/montmorillonite clay [70,71], AlCl_3_/chitosan/polyvinyl alcohol/bentonite clay [72], chitosan/hydroxyethyl cellulose/CuO [73], polylactic acid/chitosan/cellulose/ethyl lauroyl arginate [74,75], polyvinyl alcohol/cellulose/nanowhiskers/chitosan [76], chitosan/garlic/CdO-TiO_2_ [77], and poly ortho aminophenol/chitosan/metal/organic frameworks [78]. The gas barrier property, mechanical, antimicrobial, and physical characteristics are suitable for food packaging. The gas barrier property is often considered as the most important factor to ensure the shelf life of food products. The effect of MOSP in composites on properties, such as mechanical, and antimicrobial activity was investigated. CS/TA/MOSP biocomposites for food packaging applications are currently being developed and investigated.

In this study, a biocomposite film was created using solution casting, with the aim of using it for food packaging. The chemical composition, crystallinity, thermal stability, and morphology of the biocomposite film made from TA and the distinguished wt.% of MOSP were analyzed. The biocomposite film was then evaluated using different techniques, including FTIR, XRD, SEM, TGA, mechanical testing, and permeability tests for O_2_ and H_2_O vapor. Finally, the effectiveness of the film was tested as a packaging material for strawberries. This study aimed to determine how MOSP concentration affected the CS/TA biocomposite film and to evaluate the effectiveness of the film as a food packaging material. This research provides new insights into the use of biocomposite films made from TA and MOSP in food packaging and their potential as a sustainable alternative to traditional plastic packaging.

## 2. Materials and Methods

### 2.1. Materials

Low-molecular-weight (50,000–190,000 Da) chitosan was purchased from Sigma-Aldrich, Incheon, Republic of Korea. Moringa oleifera seeds were purchased from a supplier at Moringa Promise Wellness, Theni, Tamil Nadu, India. Glacial acetic acid, glycerol, methanol, and other solvents were purchased from Sigma-Aldrich in Incheon, Republic of Korea. All processes utilized double-distilled water. Without extra purification, all chemicals were utilized as received.

### 2.2. Preparation of Moringa Oleifera Seed Powder (MOSP)

**Figure 1:**
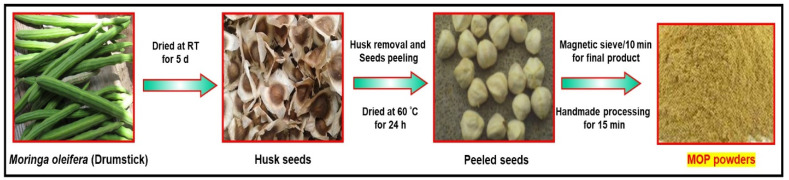
Preparation scheme of Moringa oleifera seed powder (MOSP).

Figure 1 shows the process of the development and processing of the MOSP from the Moringa oleifera seed. At 60 °C, it took 24 h for the seeds to be dried. With handmade processing (Preethi Power One Blend—MG 191, 500 W), the dried seeds were peeled and crushed into powder for 15 min. The powder that was obtained was sieved for 10 min in a magnetic sieve with mesh #200 (75 µm) used as an opening for the powder size separation. MOP was given to the powder samples.

### 2.3. The Fabrication of the Chitosan-Based Composite Film

**Figure 2:**
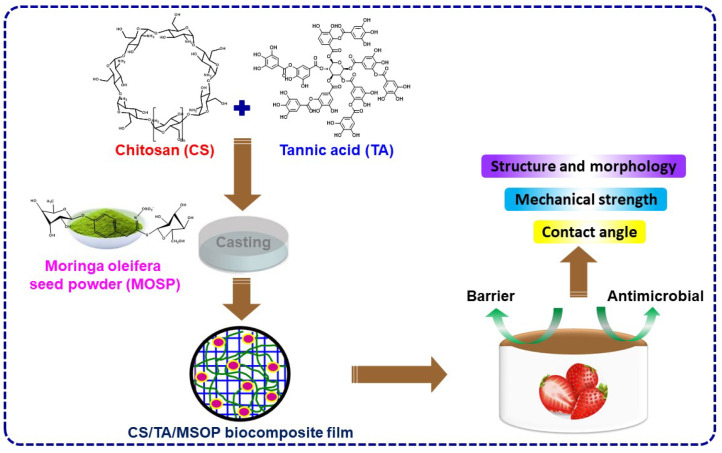
The schematic representation of the fabrication of CS/TA/MOSP biocomposites.

The diagram of the CS and CS/TA/MOSP biocomposites’ fabrication is shown in Figure 2. The solution casting method, as described earlier [79], was used to fabricate the CS/TA and CS/TA/MOSP biocomposite film. Chitosan (2.0 ± 0.1 g) was dissolved in 50.0 mL of distilled water, and then 100 ± 0.1 mg of glycerol was added. After adding the chitosan, tannic acids and MOSP (30.0 and 1.0, 3.0, 5.0, and 10.0 wt.%, respectively) were dissolved in distilled water. The uniform film-forming solution (FFS) was produced by adding 2.5 mL of 2% acetic acid and stirring the solution constantly at 80 ± 5 °C for 1 h using a magnetic stirrer. The neat CS film was also produced with acetic acid without TA and MOSP. After that, the FFS was transferred into a glass Petri dish. It was allowed to dry normally for a full night before it was baked at a temperature of about 40 °C for 24 h. Using a spatula, the dried film was removed from the Petri dish and placed in an airtight plastic bag prior to being stored at room temperature for further study. The CS/TA/MOSP biocomposite film sample nomenclature, along with details, are displayed in Table 1.

### 2.4. Characterization

The chemical structure of the CS/TA/MOSP biocomposite film was mainly identified with Fourier-transform infrared spectroscopy (FTIR) (Perkin-Elmer Spectrum Two, Waltham, MA, USA). The use of XRD analysis and measurements via a diffractometer (Rigaku, PANALYTICAL, Tokyo, Japan) was performed to study the peak pattern of chitosan and MOSP as well as shifts in the peaks after mixing the films. Scanning electron microscopy (SEM) (Hitachi, S-4800, Tokyo, Japan) was employed to study the CS/TA/MOSP biocomposite film. The thermal stability and degradation of the fabricated CS/TA/MOSP biocomposite film were studied with thermogravimetric analysis (TA Instruments, SDT Q6000, New Castle, DE, USA). According to the ASTM D3985 [80], standard procedure, the oxygen transmission rate (OTR) results for chitosan and its composite film were determined via Noselab, ATS, Bovisio-Masciago, Italy, at room temperature. As per ASTM F1249-90 [81], the water vapor transmission rate (WVTR) of the chitosan composite film was calculated with a Lyssy L80-5000 at 23 °C. The sessile drop method was applied with the contact angle instrument (Dataphysics, OCA-20, Filderstadt, Germany) to estimate the contact angle of the CS/TA/MOSP biocomposite film.

#### 2.4.1. Evaluation of Mechanical Characteristics

The CS/TA/MOSP biocomposite film’s tensile strength and elongation at break were evaluated with a universal testing machine analyzer (3345, Instron, Norwood, MA, USA), and the thickness of the composite film was calculated via a spiral distance meter (Mitutoyo micrometer, Tokyo, Japan).

Tensile strength (TS): The TS, which also demonstrates the material’s resistance to fracture, reveals its capacity to withstand more uniform plastic deformation. The equation is written below:

TS (MPa) = Fmax/(L × W)(1)

where Fmax is the maximum tensile force (N), L is the film thickness (mm), and W is the film width (mm).

Elongation at break (EAB): The major analysis to measure if the film distortion is stable or consistent is the elongation at break (EAB g/100 g). Following is an explanation of the formula:

EAB (%) = L_1_ − L_2_/L_0_ × 100 (2)

where L_0_ is the length of the film at the start; L_1_ and L_2_ are the length of the break in the film (in millimeters).

#### 2.4.2. Swelling Property Test

The composite film samples are heated and dried in an oven for a specific period before being placed in a desiccator. After fast cooling, they were measured. The films remained immersed in water for over 24 h. Drawn-out samples are weighed after drying with a lint-free cloth. A weight percentage increase is utilized to represent the absorption of water.

Swelling property (%) = Wet weight − Dry weight/Dry weight × 100(3)

#### 2.4.3. Water Solubility Test

The film segments (1 × 1 cm) were placed in 100 mL of distilled water and stirred for an hour at room temperature in a mechanical shaker in addition to evaluating the film solubility. The extra materials were filtered and dried at 110 °C for a few hours. After the samples were dried out, their final dry weight was calculated [82].

Water solubility (g/100 g) = Initial dry weight − Final dry weight/Initial dry weight × 100(4)

#### 2.4.4. Antimicrobial and Antifungal Activity

Prior to the antimicrobial test, several concentrations of MOSP were used in the CS/TA/MOSP biocomposite film, which were then cut to a similar size and uniform. The antibacterial activity of the films was evaluated against the Gram-positive *Staphylococcus aureus* and the Gram-negative *Escherichia coli* bacteria. Meanwhile, the antifungal activity was tested on the *Candida albicans* fungus with the agar disc diffusion method. To sterilize the sample film, it was placed under UV light for 30 min. A total of 200 µL of bacterial culture medium was added to 8 mL of semi-solid medium in a centrifuge tube, and it was then cooled down before it was transferred to the culture plates. The CS/TA/MOSP composite film was placed on the surface of the medium after solidification and then was afterward incubated for 24 h at 37 °C. The diameter of the zone of inhibition was then determined to calculate the rate of bacterial growth.

#### 2.4.5. Food Quality Test

The efficacy of the CS/TA/MOSP biocomposite film in protecting strawberry fruits against spoiling was examined. Fresh strawberries were cleaned prior to wrapping in the following films: (i) commercial polyethylene film and (ii) CS/TA/MOSP biocomposite film. All of these were placed on a clean plate and ignored after that. The strawberry fruits were evaluated with a five-person peer group according to their texture and color after the first and twelfth day of preservation.

#### 2.4.6. Statistical Analysis

The statistical value was calculated with an ANOVA in SPSS-21 (IBM, New York, NY, USA). To calculate the statistical differences, a one-way variance calculation was utilized, and a maximum value of *p* ≤ 0.05 was used.

## 3. Results and Discussion

### 3.1. Characterization of Moringa Oleifera Seed Powder (MOSP)

The structure of the processed MOSP was studied using FTIR analysis. The spectra (Figure S1) showed peaks in the 4000–400 cm^−1^ range. The alcohol or phenol peak, which is located at 3460 and 2980 cm^−1^, is suggestive of phenol. This functional group is abundant in the protein and fatty acid structures identified in Moringa oleifera seeds. NH_3_ elongation is shown by the peak below 3400 cm^−1^ [83]. The quantity of CH vibration is associated with the band at 2740 cm^−1^, and the presence of long carbon chain compounds, like the methylene groups in lipid and cellulose molecules, is related to these bands’ high intensity. The presence of bands between 1780 and 1450 cm^−1^, observed in Figure S1, is associated with protein and amino acids. The peak at 1740 cm^−1^ represents carboxylic groups. The band between 1660 and 1510 cm^−1^ provides the stretching vibration added to the carbonyl group (C=O). The XRD diffractograms of MOSP powder are displayed in Figure S2. The seeds include significant quantities of protein, oil, and cellulose. The plane (002) corresponds to the distinct XRD peak at 22.5°, and the amorphous region has been confirmed at 12.5° [84]. As seen in Figure S3A, the optical microscope analyzed the morphology and uniformity of the MOSP. When the particle diameter was calculated with the Image J software (version 2.1.0), the average diameter was 61.2 ± 2.3 µm. The SEM micrographs of the MOSP are shown in Figure S3B,C. This material has a porous matrix and is homogeneous [85]. According to Shirani et al. [86], porosity increases the biopolymer’s performance in reducing fungi and bacteria. Figure S4 shows the MOSP TG-DSC curve. The sample undergoes three main weight loss events: the first is associated with the loss of water, volatile compounds, and compounds with low molar mass and occurs at 133 °C. The second reason relates to the amine groups in protein producing the leakage of gases such as CO_2_ and NH_3_. The final occurrence will occur at a temperature region of 300 to 425 °C, which corresponds to MOSP.

### 3.2. Characterization of CS/TA/MOSP Biocomposite Film

**Figure 3:**
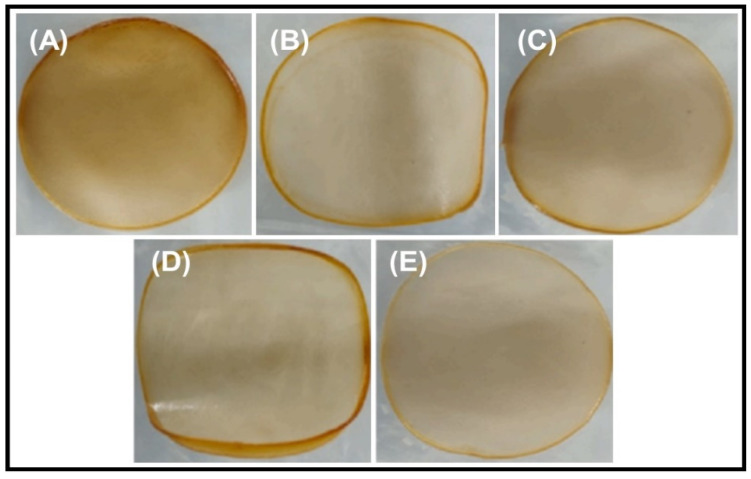
The images of the CS/TA/MOSP biocomposite films: (**A**) chitosan, (**B**) CTM-1, (**C**) CTM-2, (**D**) CTM-3, and (**E**) CTM-4.

Figure 3 presents images of the chitosan and the CS/TA/MOSP biocomposite film. Chitosan/TA and MOSP perform effectively because the film surface is almost identical to the surface of the chitosan film. FTIR, XRD, and SEM studies have been performed on the fabricated CS/TA/MOSP biocomposite film. In this section, the results of mechanical strength, thermal stability, O_2_ and H_2_O vapor permeability, water contact angle, antimicrobial activity, and food quality characteristics are presented.

#### 3.2.1. FTIR Analysis

**Figure 4:**
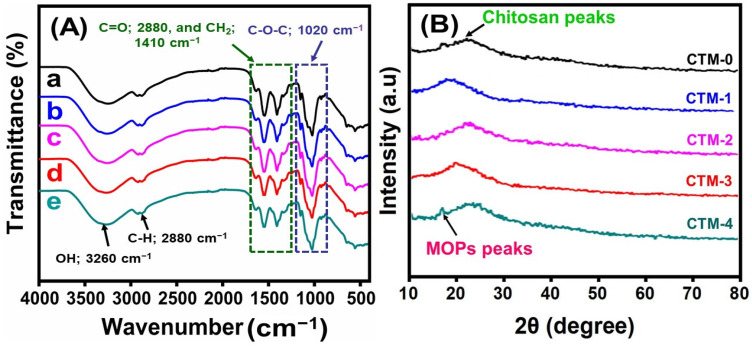
(**A**) FTIR spectrum of CS/TA/MOSP biocomposite film: (a) chitosan, (b) CTM-1, (c) CTM-2, (d) CTM-3, and (e) CTM-4; CS/TA/MOSP biocomposite film XRD patterns (**B**).

With the help of FTIR, differences in the stretching vibration and absorption peak of the CS and CS/TA/MOSP biocomposite film were studied. The CS/TA/MOSP biocomposite films and CS film infrared spectra are displayed in Figure 4A. Chitosan’s absorption peak at 3425 cm^−1^ corresponds to the stretching vibrations of NH and OH. In contrast, the absorption peaks at 2881, 1599, and 1381 cm^−1^, respectively, represent the stretching vibration of C-H, the bending vibration of the amino group, and the acetyl units. The infrared spectra were mostly the same after the addition of MOSP. However, the expansion of the link at 3425 cm^−1^ was strengthened, suggesting that the hydrogen bonding interactions had increased. The maximum levels of absorption at all three locations, 2881, 1599, and 1381 cm^−1^, were slightly replaced. The composite film did not produce a noticeable new peak when compared to the chitosan film. According to the FTIR results, MOSPs were identified in the CS-TA matrix and displayed an acceptable molecular level attachment. These results suggest that the MOSP interacted with chitosan in the interior of the films, with no effect on the surface properties of the film.

#### 3.2.2. XRD Analysis

The crystalline or amorphous nature of the film has been identified with XRD analysis. For the chitosan (CTM-0) film, a few sharp peaks can be observed in Figure 4B, indicating the presence of a material that is amorphous. The CTM-1 biocomposite film exhibited a sharper peak at 19.0°, whereas the CTM-2 biocomposite film had longer peaks, showing a reduction in the amorphous characteristics [87]. The CS film showed three prominent peaks at Bragg angles of 18.4° and 23.7°, but the CTM-1 film has a sharper peak at 19.2°. The increase in the amorphous characteristic was observed by CTM-3 and CTM-4 with larger peaks at 23.9° and 25.5°, accordingly. Pure chitosan film was shown to exhibit enhanced flexibility, surface area, and barrier characteristics, followed by an amorphous phase as indicated in the CTM-4 biocomposite film, which included more MOSP. After acting as fillers, the cohesion bonds of the matrix were enhanced to the MOSP. These results further indicate that the amorphous structure of CS has no major effect with the addition of MOSP [88].

#### 3.2.3. SEM Analysis

**Figure 5:**
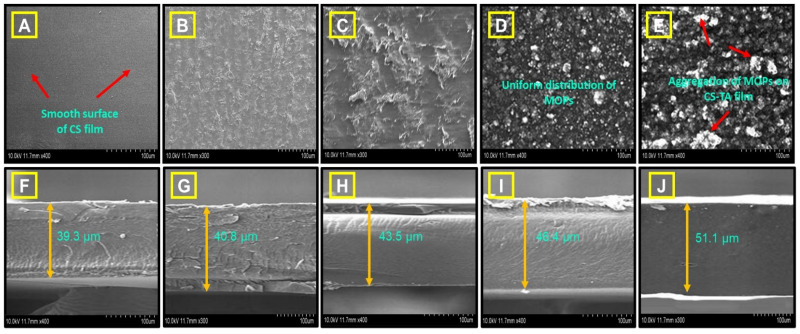
SEM images of CS/TA/MOSP biocomposite film: (**A**) neat chitosan (CMP-0), (**B**) CMP-1, (**C**) CMP-2, (**D**) CMP-3, and (**E**) CMP-4; SEM images of cross-section structure of CS/TA/MOSP biocomposite films (**F**–**J**).

Figure 5 illustrates the SEM images of the chitosan and CS/MOP composite film at 1.0, 3.0, 5.0, and 10.0 wt.%. Figure 5A shows the dispersed, instead of uniform, morphology of the pure chitosan within the film [89]. The chitosan particles were dispersed across a sizable area at random intervals. The film formed a smooth covered surface after 1.0 wt.% of MOSP was added to the chitosan polymeric composites, as indicated in Figure 5B. The film displayed an almost uniform surface with evenly mixed components [90] when we increased the concentration of the MOSP starting at 3.0 wt.%, as shown in Figure 5C. When the MOSP was raised slowly to 5.0 wt.%, the composite film showed a robust, uniform, and greater structure, as seen in Figure 5D. As shown in Figure 5E, the composite film gets a little bit denser and more compact as the MOP concentration increases to 10.0 wt.% [91]. This resulted from a powerful relationship between the carboxyl group of MOSPs and the group of amines in the chitosan matrix. Chitosan and MOSP particles were well incorporated, and as observed in Figure 5E, it was discovered that the surface of the murky wool-like particle was obstructed in the CMP-4 film. According to topographical studies, the CMP-4 film developed with chitosan, and the 10.0 wt.% of the MOSP had higher aggregation characteristics than other films.

Figure 5F–J display the cross-sections of the CS/TA/MOSP biocomposite films as SEM images. It was found that, compared to the pure chitosan sample, the addition of phenolic acids changes the structure of the films [Figure 5F]. A uniform, porous film is formed through the mixture of tannic acid with chitosan. The microstructure of the biocomposite films produced from chitosan/TA with MOSP seems more uniform and devoid of evident pores in Figure 5G–J. We believe the formation of hydrogen bonds in the chitosan structure is affected by the cross-linking effect of TA. All of the samples have a flat, crack-free surface that is smooth and uniform. We noticed small crashes in the biocomposite film of CS and CS/TA/MOSP.

#### 3.2.4. Thermogravimetric Analysis (TGA)

**Figure 6:**
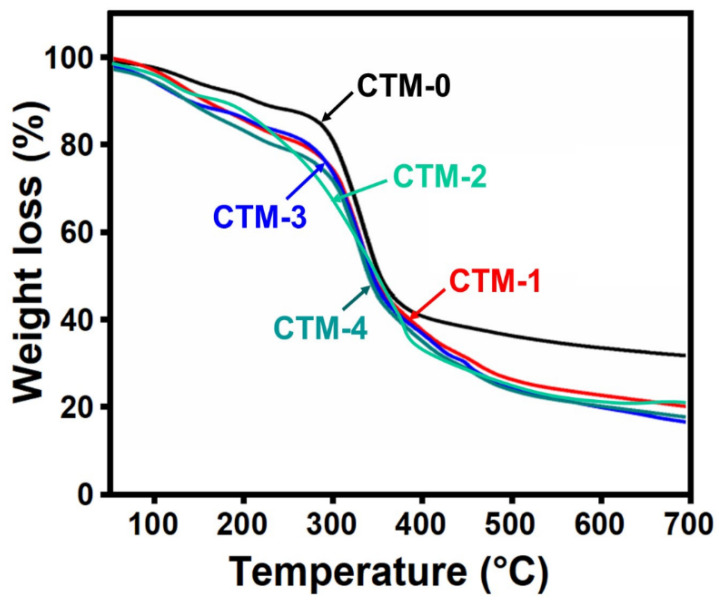
TGA curves for film samples CTM-0, CTM-1, CTM-2, CTM-3, and CTM-4 of the CS/TS/MOSP biocomposite film.

The thermal stability and decomposition of the biocomposite film fabricated from chitosan with TA and MOSP have been evaluated with TGA, and the results are shown in Figure 6. At 260.2 °C, the pure chitosan film showed an initial stage of weight loss. Water loss produced the first weight loss, which occurred at 287.1 °C; the second weight loss, which occurred at 524.6 °C, suggested that chitosan exhibited thermal degradation.

The CTM-1 film showed two instances of thermal weight loss. The first occurred at 269.43 °C, which was caused by moisture loss. The second instance occurred at 450.24 °C, where the remaining undegraded films showed a weight loss of 67.10% (the highest weight loss). This is believed to be a result of the disintegration of intermolecular and limited breakage of the molecular arrangement. The composite films of CTM-3 and CTM-4 also exhibited their weight losses at 269.1 °C and 281.0 °C, respectively, and the second weight loss at 464.0 °C and 477.2 °C, respectively, with undegraded film of 41.10 and 41.98%, respectively. Overall, the above results suggest that introducing MOSP to the MOSP has improved the thermal stability of the composite film. In comparison to chitosan, the CS-TA film composite with 1.0 wt.% MOSP showed a higher weight loss. However, Rekik et al. [92] discovered that a clean chitosan thermal degradation decreased from 240 °C to 221 °C with a 10.0 wt.% of kaolin. The following are the different chitosan/TA/Moringa oleifera (CS/TA/MOSP) biocomposite films, listed in increasing order of thermal stability:

CTM-0 ˂ CTM-2 ˂ CTM-1 ˂ CTM-3 ˂ CTM-4

#### 3.2.5. Mechanical Properties

Food packaging industries commonly utilize chitosan biocomposite film; therefore, it has to be strong and not easily fractured [93]. The measurements have been performed relating to the composite film’s tensile strength (TS) and elongation at break (EAB). With a thickness of 0.03 mm, a length of 40 mm, and a width of 6 mm, a rectangle was cut out of the film. The TS and EAB of the CS/TA/MOSP biocomposite film had major effects with the addition of MOSP, as shown in Table 2. The mechanical characteristics of the biocomposite film, including TS and EAB, showed an increase with increasing MOSP concentration.

**Figure 7:**
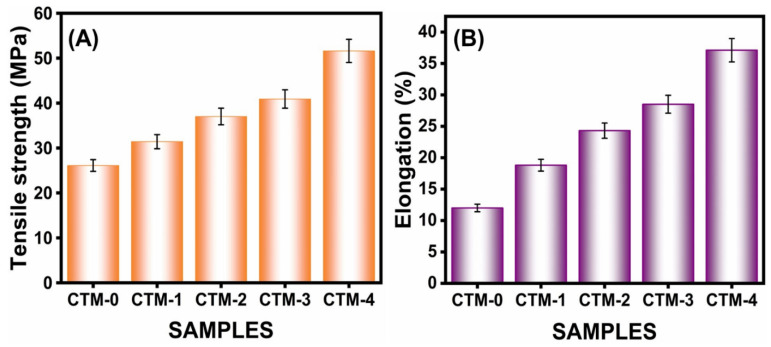
(**A**) Tensile strength and (**B**) elongation at break of chitosan, CTM-1, CTM-2, CTM-3, and CTM-4 biocomposite film. Error bars represent ± 5.01 standard errors.

The neat CS film has a TS and an EAB of 26.1 MPa and 12%, respectively. The addition of MOSP greatly improved the tensile strength. Tensile strengths of the CS/TA biocomposite film with 5.0 and 10.0 wt.% MOSP were 40.9 MPa and 51.6 MPa, respectively [Figure 7A]. The most effective loading was obtained via the addition of 10.0 wt.% of MOSP, which dispersed well in the chitosan with the TA matrix and resulted in more stress transfer from the chitosan to the filler. In addition to the increased surface area of the particles, there were greater filler/polymer matrix interfacial relationships, which enhanced the shear mechanism’s capacity to transfer stress from the chitosan matrix to MOSP. Tensile values of the biocomposite film were increased, and the load was dealt with with greater efficiency [94]. On the other hand, the chitosan/TA film showed an EAB that was 28.5% with a 5.0 wt.% of MOSP loading and 37.1% at a 10.0 wt.% MOSP loading [Figure 7B]. Chains of chitosan/TA are less flexible as a result of MOSP’s reinforcing effect. The rigidity and brittleness grew greater as flexibility reduced. Thomas et al. [89] reported similar results; they observed that the elongation at break was significantly increased when almond gum powder was added to chitosan film at a concentration of 1.0 wt.%.

#### 3.2.6. Swelling Property

**Figure 8:**
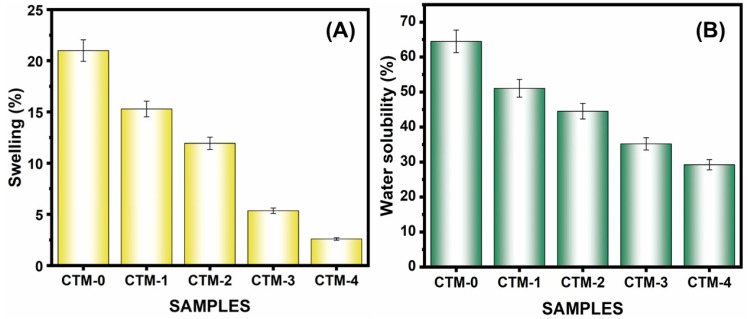
(**A**) Swelling and (**B**) water solubility of chitosan, CTM-1, CTM-2, CTM-3, and CTM-4 biocomposite film. Error bars represent ± 5.01 standard errors.

Results of a swelling property test for multiple chitosan-based film types are shown in Table 3. The chitosan film demonstrated increased swelling characteristics, as seen in Figure 8A, while the CTM-4 film with the maximum MOSP concentration showed less swelling characteristics. The result is confirmed by the observation that MOSP tends to increase the hydrophobicity of the film, leading to significantly decreased water absorption and resulting in lower swelling [95].

#### 3.2.7. Water Solubility Test

Figure 8B illustrates the evaluation of chitosan and its composite film for the water solubility test. The water solubility of the CS/TA/MOSP biocomposite film reduced noticeably as the dispersion of MOSP increased [96], as seen in Table 3. Water disintegrability was the maximum for the chitosan film and lower for CTM-4 with 10.0 wt.% of MOSP. The biocomposite film that proved to be the most water-resistant, so it was suitable for food packaging, was CTM-4.

#### 3.2.8. Oxygen Transmission Rate (OTR)

One of the most essential characteristics of the packing material was the permeability of oxygen. Table 3 presents the outcomes of the study on the permeability in biocomposites fabricated with CS, TA, and different loadings of MOSP. With respect to the presence of MOSP, the value of the oxygen transmission rate changes from 31.1 ± 1.6 to 82.6 ± 3.0 cc/m^2^/24 h. The CS film was enriched with MOSP, which enhanced its barrier characteristics. The interaction of two occurrences leads to diffusion via the CSs:

 i. The area available for gas diffusion is reduced when MOSP is introduced.
 ii. The increase in the length of the path that gaseous particles must travel to move via the film.

MOSP was added to CS/TA in the current work; however, it had no noticeable effect on the type of amorphous CS. It is believed that the MOSP is well dispersed, offering much tortuosity in the oxygen molecule diffusion manner, which is responsible for the decreased oxygen permeability of the composite film containing MOSP. After MOSP was added to it, the OTR values of the biocomposite film decreased.

#### 3.2.9. Water Vapor Transmission Rate (WVTR)

The water vapor transmission rate is a major barrier property of the CS film used in packaging. The values of the WVTR for the CS/TA/MOSP biocomposite films are presented in Table 3. MOSP was added into the CS/TA film, which notably reduced the WVTR of the CS biocomposite film. In comparison with pure CS, which possesses a WVTR value of 22.0 ± 2.5 g/m^2^/24 h, the CS/TA biocomposite’s WVTR value ranges from 18.2 ± 1.3 to 10.1 ± 2.8 g/m^2^/24 h. According to the MOSP concentration, the WVTR of the biocomposites was reduced by 6.8–61.9%. The reduced water vapor permeability of composites is induced to the even distribution of larger MOSP particles in the CS/TA matrix. The formation of MOP increased the diffusing paths by convoluting the CS’s path.

#### 3.2.10. Water Contact Angle (WCA) Analysis

**Figure 9:**
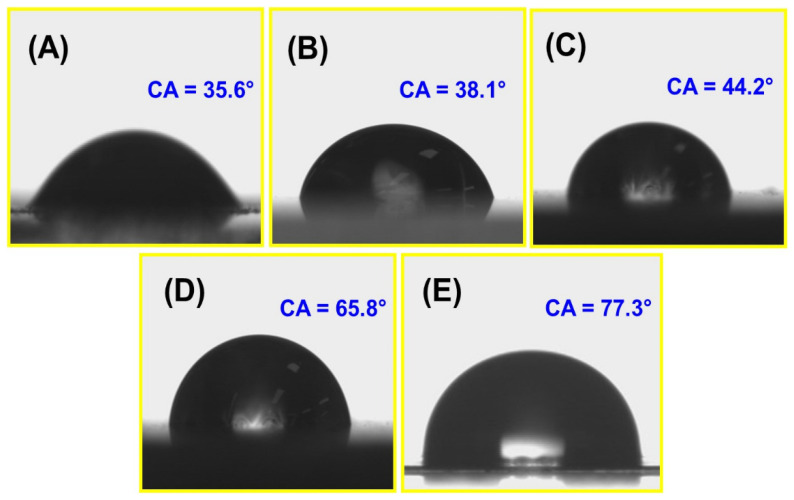
Water contact angle of fabricated biocomposite film; (**A**) chitosan, (**B**) CTM-1, (**C**) CTM-2, (**D**) CTM-3, and (**E**) CTM-4.

The surface hydrophilicity and wettability of the biocomposite film were measured using water contact angle (WCA) measurements. Figure 9 illustrates the film’s water contact angle. The chemical and structural characteristics of the matrix are expected to influence the surface hydrophilicity of the biocomposite film. The WCA for the CS and CS/TA/MOSP biocomposite film was significantly below 90°, which rendered them hydrophilic. However, the CS combination decreased the CTM-4 film’s wettability, which in turn raised the film’s WCA. The roughness of the film increased in the subsequent order, according to morphological analyses (Figure 5), from CTM-0 to CTM-1 to CTM-2 to CTM-3 to CTM-4. The reduction in hydrophilicity occurred in the same order. The MOSP reinforcing was an influence in the films’ decreased hydrophilicity. However, the WCA and hydrophobicity of the CTM-4 biocomposite film greatly improved as a result of the surface’s enhanced roughness. The improvement in the hydrophobicity of a biocomposite film when using MOSP can be attributed to the presence of certain compounds in the Moringa oleifera. MOSP is rich in various phytochemicals, including fatty acids, phenolic compounds, and proteins. Some of these compounds have hydrophobic characteristics, which can contribute to reducing the water affinity of a film. Moringa oleifera seed powder has cross-linking properties, influencing the overall integrity of the film. Cross-linking can enhance the hydrophobicity of the film by reducing its porosity and improving its resistance to water penetration. Similar results were noted for the composite film produced from chitosan and almond gum. The results revealed that the increased loading of almond gum powder resulted in enhanced surface roughness and hydrophobicity [97]. With the goal to enhance the performance of biocomposite films in a wide range of applications, such as packaging, coatings, and biomedical materials, manufacturers frequently study plant-based materials like Moringa oleifera.

#### 3.2.11. Antimicrobial and Antifungal Activity

**Figure 10:**
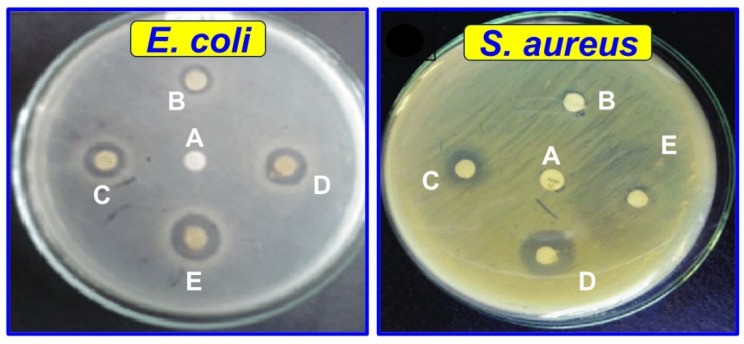
Antimicrobial activity for *E. coli* and *S. aureus* of CS/TA/MOSPs biocomposite films; (A) chitosan, (B) CTM-1, (C) CTM-2, (D) CTM-3, and (E) CTM-4.

As seen in Figure 10, the inhibition zone methods were used to determine the antimicrobial properties of the biobased CS/TA/MOSP biocomposite films. As shown in Table 4, the antimicrobial activity of the different composite films was measured against the bacteria *E. coli* and *S. aureus*. As a result of this study, a chitosan (CTM-0) film with a 30.0 wt.% of TA and a biocomposite film (CTM-4) with a 10.0 wt.% of MOSP showed strong antimicrobial properties when compared to other films that had either minimal or no MOSP. This was because MOSP can interact with the chitosan/TA film owing to its network structure, which contacts bacterial walls to inhibit microbial activity. The antifungal activity of the CS/TA/MOSP films was tested against fungi (*Aspergillus niger*, and *Candida albicans*) by the agar diffusion method. As shown in Figure S5, the addition of MOSP significantly elevated the antifungal activity of the CTM-4 films.

**Figure 11:**
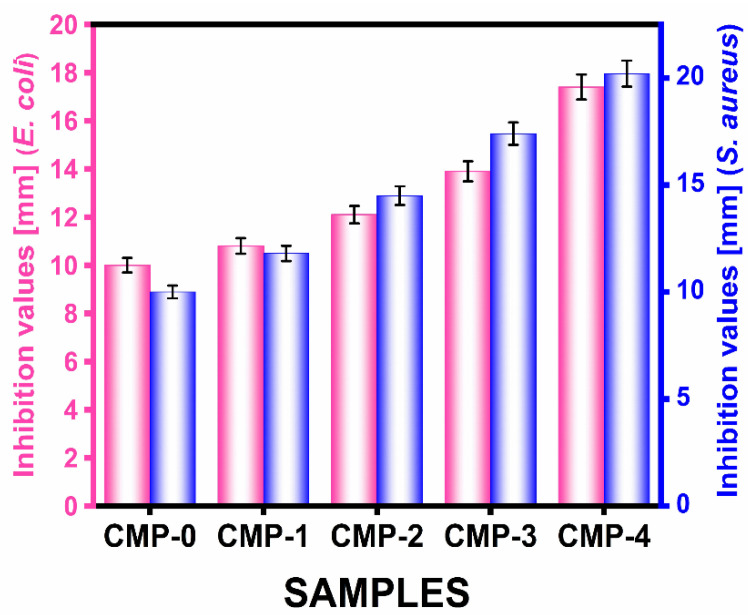
Inhibition values for *E. coli* and *S. aureus* of CS/TA/MOSP biocomposite film.

The results of a study and photograph of the CS/TA/MOSP biocomposite film’s inhibition values for *E. coli* and *S. aureus* are presented in Figure 11. The CTM-4 film had higher antimicrobial activity than CS and another film at the MOSP concentration, showing that MOSP antimicrobial properties are sufficient. Once a clear zone formed around the film disk on the media plate, it showed proof of inhibition against a particular bacterium. When the whole zone area was calculated and reduced from the disk area, the zone of inhibition was identified. The inhibition rates for both bacteria are above 90% when the MOSP concentration is equal to or more than 5.0 wt.%, which fulfills the minimal criteria for food packaging plastics.

#### 3.2.12. Shelf-Life Analysis

**Figure 12:**
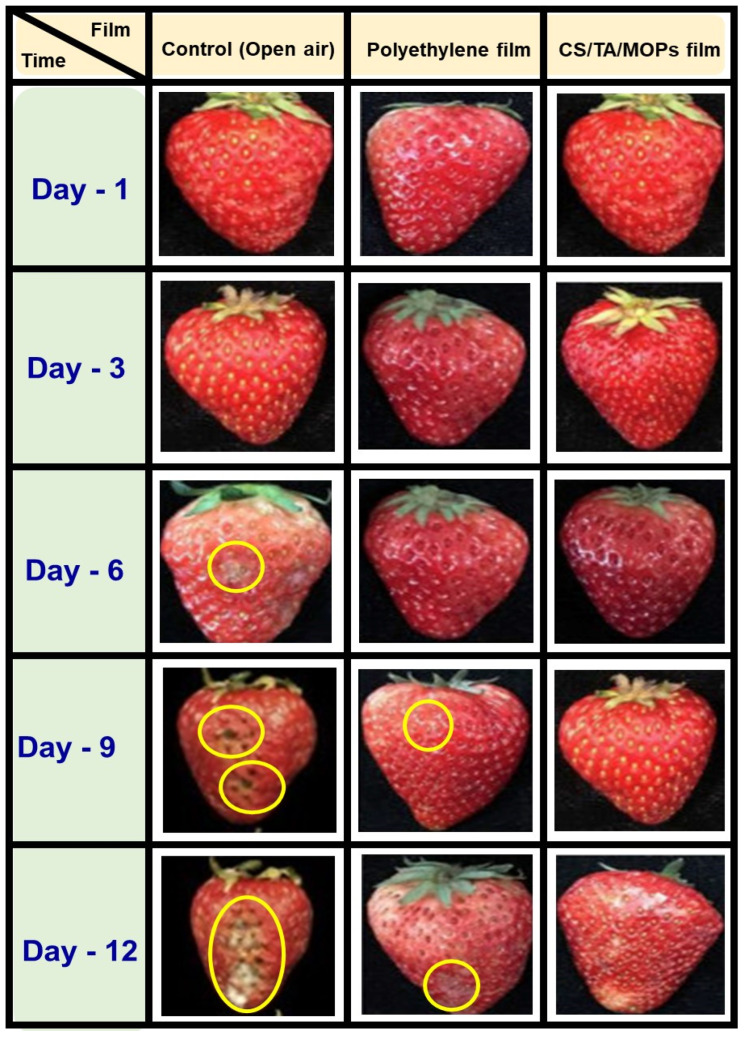
Images of food quality test using strawberries with open-air, commercial polyethylene film, and CS/TA/MOSP biocomposite film during storage at room temperature.

The major reason for a decrease in strawberry fruit quality and its limited shelf life is the growth of bacteria. The shelf life of unpreserved strawberries—between the second and sixth days—decreases their marketability and limits their chance of profit. The fabricated CS/TA/MOSP biocomposite film has antimicrobial activity that can provide a favorable environment within the packaging, preventing the growth of bacteria in strawberries and extending their shelf life. As shown in Figure 12, the test was performed in addition to commercial polyethylene-based packaging film and open-air packing for a duration of 12 days. On the ninth day of observation, microbial growth was seen and was visible in wrapped packaging of commercial polyethylene. The bacterial zone increased over time, reaching a 100 g/100 g zone on the 12th day of observation. However, in the case of fabricated antimicrobial (CS/TA/MOSP)-based packaging, no discernible microbial growth was seen. On the third day of observation, there were evident shape changes in the strawberry in the case of open-air packing. The open strawberry fruit had a leftward slope and shrank in size as a result of moisture loss. Open strawberry fruits grew fungus and were no longer suitable for human consumption.

Figure 12 represents the color changes that were noticed after the fruit of the strawberry had been stored for 12 days at room temperature, initially in open-air, commercial polyethylene film and then in CS/TA/MOSP biocomposite film. When in open air, the strawberry fruit took on a distinct form and changed color after it was handled outside; after being wrapped in commercial polyethylene film, the strawberry fruit acquired an evident mildewed appearance and formed into a spot. The yellow circle on the strawberry fruits illustrates a few fungal growths in the polyethylene film-covered strawberry fruit. As demonstrated in Figure 12, an excellent interaction developed within the MOSP and biobased chitosan, enhancing the CTM-4 film performance and showing the packages covered from the commercial polyethylene film. With the 10.0 wt.% of MOSP in the chitosan of the CS/TA film, the distributed MOSP prevented the growth of bacteria. In the study conducted by Priyadarshi et al. [98], it was found that increasing the particle content in chitosan film led to higher transparency, resulting in better food preservation and protection from light. The previous study also confirmed the potential application of the packages formed from the chitosan with 10.0 wt.% of TiO_2_ [99]. In addition, the chitosan composite film can be used as a non-toxic and suitable shield to protect food crops from contaminants. Therefore, the recently developed CTM-4 biocomposite film is more suitable for food packaging.

## 4. Conclusions

A biocomposite film was prepared using the sol cast process, which consisted of chitosan, tannic acid, and MOSP. MOSP, which has both particulate and spherical shapes, was evenly distributed in chitosan/TA. The addition of MOSP into chitosan/TA resulted in an improvement in the mechanical, barrier, and antimicrobial activity of the film. The functional groups present in the film can be identified through FTIR. The presence of MOSPs made the biocomposite film amorphous, which was revealed in XRD analysis. The MOSP in chitosan/TA was uniform and evenly dispersed, as shown in the SEM images. The antimicrobial activity test results demonstrated that the CS/TA/MOSP (CTM-4) biocomposite film had superior antimicrobial activity compared to pure CS and other biocomposite films (CTM-1, CTM-2, CTM-3). This suggests that the MOSP and TA biocomposites improved the antimicrobial activity. Based on the results of food quality tests, the CS/TA/MOSP biocomposite film extended the shelf life of strawberries from 1 to 12 days, surpassing that of pure chitosan film. This indicates that the CS/TA/MOSP biocomposite film has the potential for use in food packaging. Overall, this study presents a composite film for food packaging with sufficient antimicrobial activity.

## Figures and Tables

**Table 1:** Material formulation in blends and composite film preparation.

S. No.	Formulated Films	Nomenclature	CS (wt.%)	TA (wt.%)	MOSP (wt.%)
1.	Chitosan (100.0)	CTM-0	100.0	--	--
2.	CS/TA/MOSP (69.0:30.0:1.0)	CTM-1	69.0	30.0	1.0
3.	CS/TA/MOSP (67.0:30.0:3.0)	CTM-2	67.0	30.0	3.0
4.	CS/TA/MOSP (65.0:30.0:5.0)	CTM-3	65.0	30.0	5.0
5.	CS/TA/MOSP (60.0:30.0:10.0)	CTM-4	60.0	30.0	10.0

**Table 2:** Mechanical properties of the biocomposite films fabricated from chitosan/TA and MOSP.

S. No.	Samples	Tensile Strength (MPa)	Elongation at Break (wt.%)	Solubility in Water (wt.%)	Swelling (wt.%)
1.	CTM-0	26.1 ± 1.6	12.0 ± 2.5	64.52	21.00
2.	CTM-1	31.4 ± 1.1	18.8 ± 1.3	51.10	15.30
3.	CTM-2	37.0 ± 2.7	24.3 ± 2.2	44.54	11.94
4.	CTM-3	40.9 ± 1.0	28.5 ± 1.9	35.20	5.36
5.	CTM-4	51.6 ± 3.0	37.1 ± 2.8	29.25	2.60

**Table 3:** OTR and WVTR values of chitosan and CS/TA/MOSP biocomposite film.

S. No.	Samples	OTR (cc/m^2^/24 h)	WVTR (g/m^2^/24 h)
1.	CTM-0	82.6 ± 3.0	22.0 ± 2.5
2.	CTM-1	71.9 ± 1.0	18.2 ± 1.3
3.	CTM-2	51.0 ± 2.7	16.3 ± 2.2
4.	CTM-3	43.4 ± 1.1	12.5 ± 1.9
5.	CTM-4	31.1 ± 1.6	10.1 ± 2.8

**Table 4:** Antimicrobial activity of chitosan and CS/TA/MOSP biocomposite film.

Microorganisms/Samples	Antimicrobial Activity [Zone of Inhibition (dia. in mm)]				
	CTM-0	CTM-1	CTM-2	CTM-3	CTM-4
*E. coli*	10.0 ± 1.05 ^a^	10.8 ± 2.50 ^c^	12.1 ± 3.31 ^c^	13.9 ± 1.10 ^a^	17.4 ± 3.05 ^b^
*S. aureus*	10.3 ± 2.84 ^c^	11.8 ± 3.15 ^b^	14.5 ± 1.85 ^b^	17.4 ± 1.00 ^a^	20.2 ± 2.22 ^c^

The mean ± standard deviation of three replicates is used to express the results. a–c: Distinct letters in the same column denote statistically significant changes within film samples (*p* < 0.05).

## Data Availability

Data are contained within the article.

## Supplementary Materials

The following supporting information can be downloaded at: https://www.mdpi.com/article/10.3390/polym16070937/s1, Figure S1 FTIR spectra of the *Moringa oleifera* seed powder (MOPS). Figure S2 XRD patterns of the MOSP. Figure S3 (A) Optical microscopy image; (B, and C) Scanning electron microscopy images of the MOSP. Figure S4 TG-DSC curves of MOSP. Figure S5 Antifungal activity for *A. niger* and *C. albicans* of CS/TA/MOSPs biocomposite films; (A) Chitosan, (B) CTM-1, (C) CTM-2, (D) CTM-3, and (E) CTM-4.
