# Supplementary material for: Chitosan-Based Films Blended with Tannic Acid and Moringa Oleifera for Application in Food Packaging: The Preservation of Strawberries (Fragaria ananassa)
Source: Polymers (Basel). 2024 Mar 29;16(7):937. doi: 10.3390/polym16070937 (PMC11013215; doi:10.3390/polym16070937)
Supplement: Supplementary file 1 [file polymers-16-00937-s001.zip › polymers-2921314-supplementary.pdf]

Supplementary material for:

Chitosan-based Films Blended with Tannic Acid and *Moringa oleifera* for Application in Food Packaging: The Preservation of Strawberries (*Fragaria ananassa*)

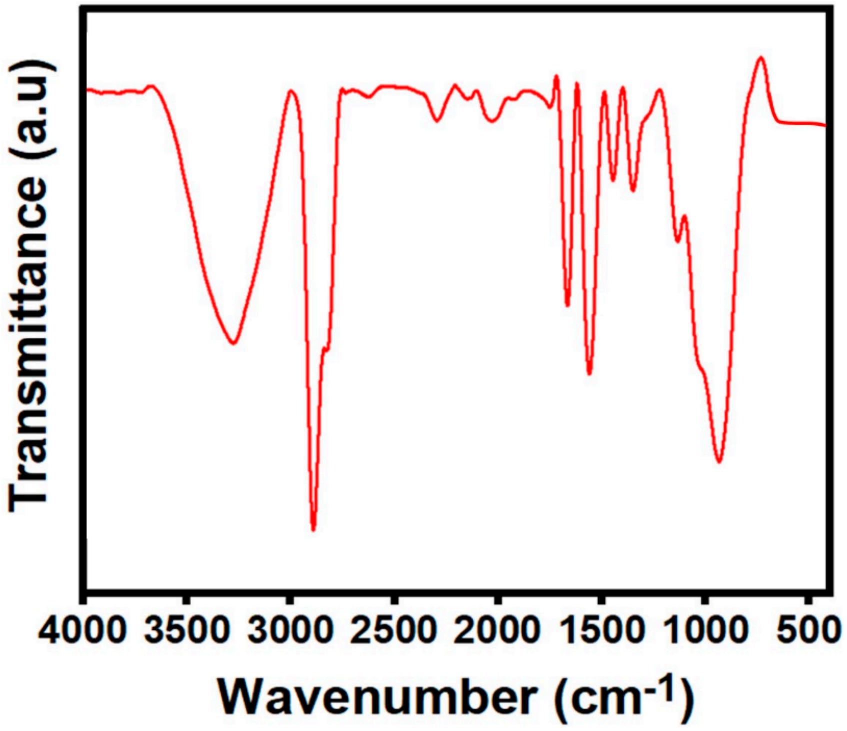

Figure S1. FTIR spectra of the *Moringa oleifera* seed powder (MOPS).

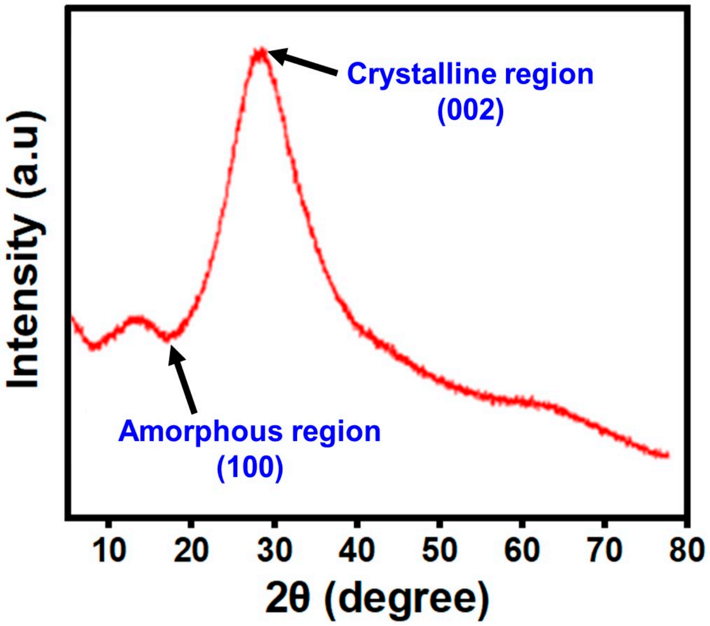

Figure S2. XRD patterns of the MOSP.

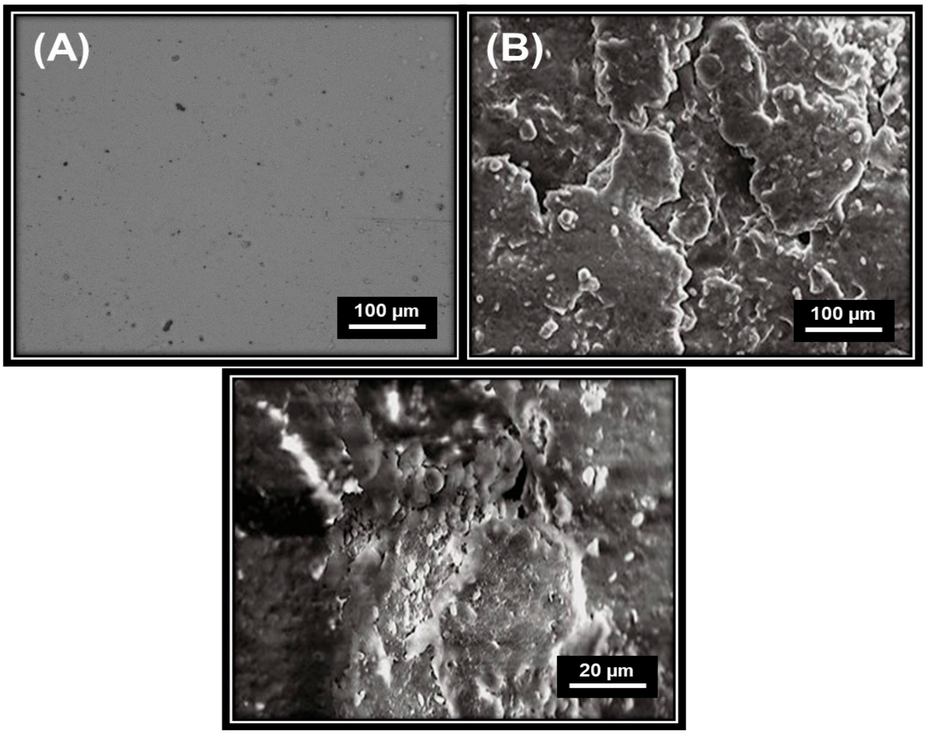

Figure S3. (A) Optical microscopy image; (B, and C) Scanning electron microscopy images of the MOSP.

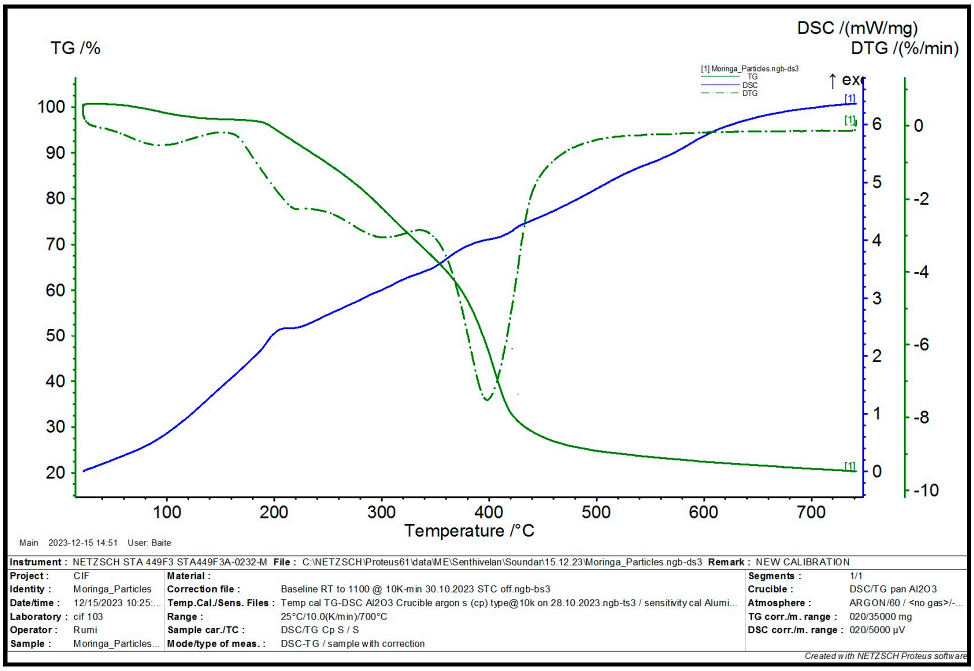

Figure S4. TG-DSC curves of MOSP.

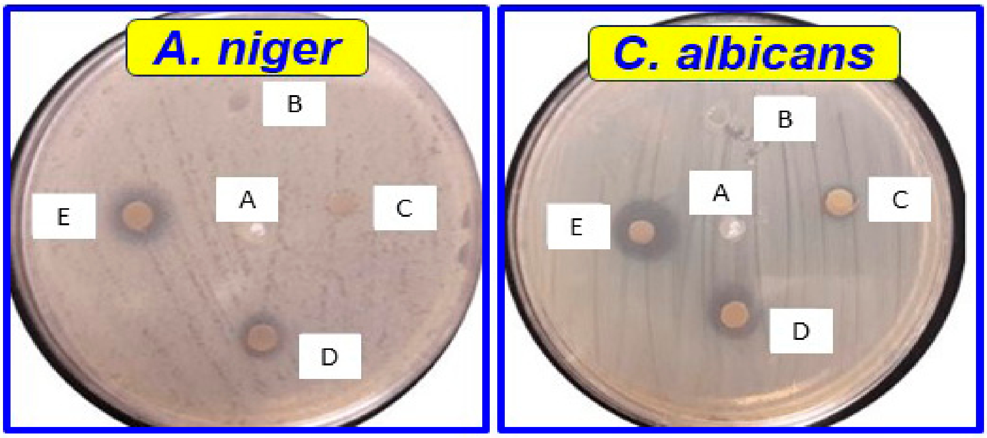

Figure S5. Antifungal activity for *A. niger* and *C. albicans* of CS/TA/MOSPs biocomposite films; (A) Chitosan, (B) CTM-1, (C) CTM-2, (D) CTM-3, and (E) CTM-4.
